# Microscopic Observation of Proliferative Membranes in Fibrocontractive Retinal Disorders

**DOI:** 10.1155/2019/9647947

**Published:** 2019-07-28

**Authors:** Rino Frisina, Francesco Tessarolo, Ivan Marchesoni, Federico Piccoli, Emiliana Bonomi, Patrizio Caciagli, Edoardo Midena, Giandomenico Nollo

**Affiliations:** ^1^Department of Ophthalmology, University of Padova, Via Giustiniani 2, I-35128 Padova, Italy; ^2^Department of Industrial Engineering, University of Trento, Via Delle Regole 101, I-38123 Mattarello, Trento, Italy; ^3^Healthcare Research and Innovation Program (IRCS-PAT), Bruno Kessler Foundation, Via Sommarive 18, I-38123 Trento, Italy; ^4^Multizone Unit of Ophthalmology of Autonomous Province of Trento, Corso Verona 6, I-38123 Rovereto, Trento, Italy; ^5^Department of Laboratory Medicine, Azienda Provinciale per i Servizi Sanitari, Largo Medaglie d'Oro 9, Trento, Italy

## Abstract

Proliferative membranes of fibrocontractive retinal disorders are extensively studied from the morphological and evolutive point of view. Despite this, little is known of their cellular composition. In this study, the authors investigated the morphological characteristics and cell composition of various types of surgically excised proliferative membranes and internal limiting membranes (ILMs), in order to provide new data supporting or challenging the pathogenic theories proposed until now. Sixty-nine specimens from 64 eyes of 64 consecutive patients were collected at surgery and subjected to a multilevel analysis by means of optical and electron microscopy. Membrane samples were semiquantitatively evaluated for the amount and distribution of cell nuclei and pigment. Immunohistochemical staining was performed with antibodies to alpha smooth muscle actin and CD68. Data were analyzed after grouping according to the following tissue types: ILM (20 specimens), epiretinal membrane (ERM) (22 specimens), ILM + ERM (20 specimens), and proliferative vitreoretinopathy (PVR) (7 specimens). The cell components found in the ERM specimens, like myofibroblasts, macrophages, and polymorphonuclear cells, were recognized as the expression of cell migration and differentiation that induced an inflammatory process and a fibroproliferative repair process. The detection of pigments in specific types of ERM, like those associated with lamellar macular hole (LMH) or secondary to retinal detachment (RD), diabetes, and PVR, suggested that retinal pigment epithelium (RPE) cells may have a role in the development of these vitreoretinal disorders. The reduction of the ERM cellularity with the patient's age supports the hypothesis that ERM evolves in time up to a fibrous tissue formation.

## 1. Introduction

Epiretinal membrane (ERM) formation is a well-known disease entity that can be idiopathic or secondary [1]. Idiopathic ERM (iERM) occurrence, causing a macular pucker (MP), usually is an age-related process [2, 3]. Secondary ERM can be due to a variety of retinal disorders including proliferative diabetic retinopathy (PDR), central retinal vein occlusion (CRVO), uveitis, proliferative vitreoretinopathy (PVR), retinal detachment (RD) surgery, trauma, macular hole (MH), retinitis pigmentosa, myopia, Terson syndrome, Eales disease, and Coats disease [4–9]. ERM can be distinguished in three types based on their cell composition and closely associated with the underlying disease: conventional or tractional ERM (C ERM) commonly associated with MP; atypical ERM (A ERM) associated with full thickness macular hole (FTMH) and lamellar macular hole (LMH), also known by various names: “dense,” “epiretinal proliferation,” “degenerative ERM” [10–14]; and neovascular ERM associated with metabolic or vascular retinal diseases. Although the pathogenic mechanisms underlying the different vitreoretinal disorders have been widely studied, the types of cells involved in these processes are not yet fully understood. Studies on excised tissue obtained during surgical treatment of different fibrocontractive retinal disorders have demonstrated that the number and types of cells found varies considerably. A wide variety of cell types such as glial cells (including microglia, Müller cells, and fibrous astrocytes), cells from the retinal pigment epithelium (RPE), blood-borne immune cells (macrophages, lymphocytes, and neutrophils), fibrocytes, and myofibrocytes were identified [15–19]. The aim of this study is to evaluate the microscopic characteristics of vitreoretinal membranes surgically excised for treating different types of fibrocontractive retinal disorders. In this study, the authors investigated the microscopic characteristics and cell composition of a range of surgically excised proliferative membranes and internal limiting membranes, in order to provide new data supporting or challenging the pathogenic theories proposed until now.

## 2. Materials and Methods

Intraocular membrane tissue specimens were collected during a consecutive series of vitreoretinal surgeries for the treatment of patients affected by different fibrocontractive retinal disorders. All interventions were performed by the same surgeon (R. F.) at the Department of Ophthalmology in Rovereto, Trento, Italy, between January 2016 and September 2017. Ethics Committee approved this study. At the time of surgery, each participant was informed about the benefits and potential risks of the treatment. Informed consent was obtained from all participants. Collected data included age (years), gender (male/female), eye (right/left), underlying pathology causing surgical treatment, and best corrected visual acuity (BCVA) measured in Snellen and converted to the logarithm of the minimum angle of resolution (logMAR). Specimens were catalogued according to the type of excised tissue according to surgeon indication: internal limiting membrane (ILM), ERM, ERM + ILM when both membranes were excised simultaneously, and PVR. In addition, ERM specimens were grouped according to the recently proposed tomographic criteria for the definition of ERM associated with LMH: conventional ERM (C ERM) and atypical ERM (A ERM) [12, 14]. C ERM was characterized by a highly reflective line adjacent and overlying the retinal nerve fibre layer, and A ERM was characterized by a thick membrane delimited by a highly reflective line and filled by the moderately reflective material [12, 14].

### 2.1. Preparation of Specimens

Excised tissues were deposited in between silicon foam foils, inserted in a histology cassette, labelled, and immediately fixed in 10% buffered formalin for a minimum of 24 hours before proceeding with the microscopic preparation and analysis. Fixed membranes were prepared for several different microscopic analyses with both optical microscopy (OM) and scanning electron microscopy (SEM). All samples were washed twice in distilled water and whole mounted on glass slides for a first analysis of the unstained sample by OM. A set of representative images were obtained under an optical microscope (DLIM, Leica, Germany) equipped with a high-resolution CCD colour camera (DFC420, Leica, Germany), with a magnification ranging from 40 to 630 times. Colour images of unstained samples were obtained under transmitted light and phase contrast to evaluate tissue morphology and presence of pigment. Samples were then unmounted and stained with haematoxylin (5 mins in Mayer's haematoxylin) before being mounted again and observed for the presence of nucleated cells. A new set of representative colour images was obtained under the same optical microscope at similar magnification.

#### 2.1.1. Cellularity Score

**Figure 1:**
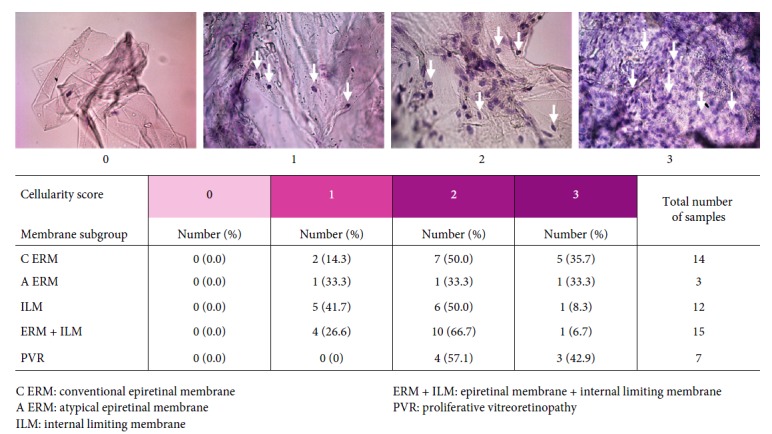
Cellularity score. Representative images for each score criteria are reported on top of the image. Cellularity score results according to each membrane specimen subgroup are summarized in the table.

OM imaging after staining with haematoxylin allowed to evaluate the presence or absence of cellular nuclei and their distribution (homogeneous or inhomogeneous) across the tissue sample. The amount of cell nuclei (cellularity) was scored semiquantitatively from 0 to 3 according to the following semiquantitative criteria: 0 (absence of nuclei), 1 (≤50 nucleated cells per mm^2^), 2 (50–100 nucleated cells per mm^2^), and 3 (≥100 nucleated cells per mm^2^) (Figure 1). The cellularity scores were assigned after joined review and evaluation of the collected images by three investigators (F. P., E. B., and F. T.).

#### 2.1.2. Pigmentation Score

**Figure 2:**
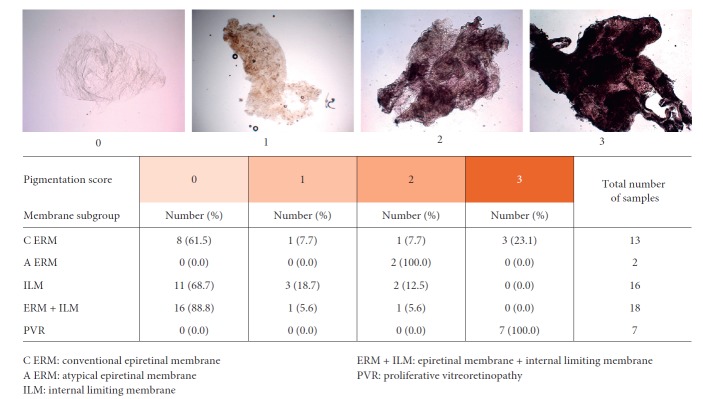
Pigmentation score. Representative images for each score criteria are reported on top of the image. Pigmentation score results according to each membrane specimen subgroup are summarized in the table.

The amount of intratissue pigment (pigmentation) was evaluated according to a semiquantitative score ranging from 0 to 3 according to the following definitions: 0 (absence of pigment), 1 (granulated pigment in <50% of the tissue sample), and 2 (granulated pigment present across the whole sample or yellow/light brown pigmented diffused areas in less than 50% of the sample) to 3 (brown pigmented areas diffused in the whole sample) (Figure 2). Similar to the cellularity, pigmentation scores were assigned after joined review and evaluation of the collected images by three investigators (F. P., E. B., and F. T.).

#### 2.1.3. Morphological Study

A subset of seventeen samples also underwent ultrastructural characterization by scanning electron microscopy (SEM). SEM analysis was conducted by using both environmental (E-SEM) and conventional high-vacuum (HV-SEM) operative modes. The E-SEM mode allowed imaging of fully hydrated samples without the need for conductive coating, thus informing the native status of the sample. HV-SEM was applied to obtain high-resolution images of micromorphologies at the membrane surface. Differently from E-SEM, HV-SEM required complete dehydration and metallic coating for guaranteeing sample conductivity. HV-SEM preparation was obtained by dehydration in ascending hydroalcoholic solutions, drying in a laminar flow cabinet, mounting on a sample holder, and sputter-coating with gold. SEM imaging in both E-SEM and HV-SEM modes was performed with a XL30 ESEM FEG scanning electron microscope (FEI, Philips, The Netherlands). High-resolution micrographs were obtained with a magnification ranging from 500 to 2000 time in the E-SEM mode and up to 10000 times in the HV-SEM mode. Details of surface morphology and any eventual cell-like structure were imaged and stored.

#### 2.1.4. Immunohistochemical Study

In a second different subset of seventeen samples, immunohistochemical staining was performed with antibody to alpha smooth muscle actin (alpha-SMA) to target intracellular actin filaments or CD68 for macrophages/microglia.

#### 2.1.5. Statistical Analysis

SPSS Software Version 22.0 (IBM Corporation, New York, NY) for the statistical analysis was used. Descriptive statistics are absolute and relative frequencies for qualitative variables (gender, eye, types of specimens, and pathology) and means, standard deviation, and median for quantitative variables (age and BCVA). The logistic regression model was used to evaluate the association between subgroup of specimens and cellularity score. The Shapiro–Wilk test was used for evaluating normal distribution of values. The ANOVA test and post hoc correction for multiple comparison (Bonferroni correction) were used for evaluating correlation between age vs. cellularity score and age vs. pigmentation score. Fisher's test was used to evaluate the association between pigmentation score and specimen subgroups.

## 3. Results

Sixty-nine specimens were collected from 64 eyes: 36 (56.25%) right and 28 (43.75%) left; of 64 consecutive patients, 28 (43.75%) were males and 36 (56.25%) were females. Patients mean age was 72.35 ± 7.06 (range from 58 to 88 years). Preoperative mean BCVA was 0.7 ± 0.2 (20/100 letters), from 0.2 to 1 logMAR. Data were analysed after grouping according to the following tissue types: ILM (20 specimens), ERM (22 specimens), ILM + ERM (20 specimens), and PVR (7 specimens). Tables 1 and 2 summarize demographic data of specimen subgroups and the surgically treated retinal diseases. The comparison among the subgroups of specimens did not highlight any statistical difference in age, gender, and eye of patients.

### 3.1. Cellularity

**Figure 3:**
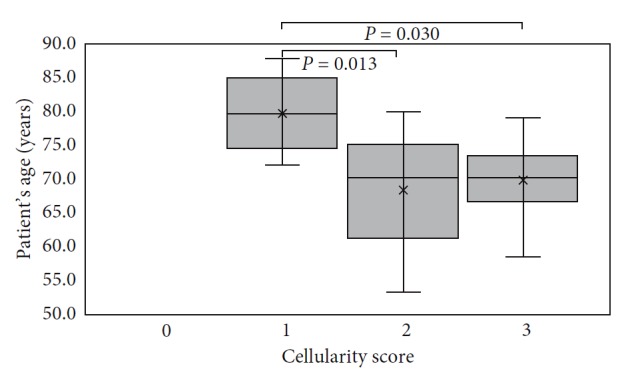
Cellularity score boxplot vs patient's age. *P* value is indicated for subgroups showing statistical difference.

The cellularity score was evaluated on 51 out of the 69 collected specimens. C ERM and PVR subgroups were characterized by higher cellularity scores than ILMs. Microscopic study showed that 85.7% of specimens of the C ERM subgroup and 100% of the PVR subgroup were in the score 2-3. ERM + ILM and ILM subgroups were characterized by low-intermediate cellularity; specifically, 93.3% of ERM + ILM specimens and 91.7% of ILM specimens were characterized by a cellularity score 1-2 (Figure 1). The A ERM subgroup resulted homogenously distributed over the full range of cellularity scores. Logistic regression analysis showed a significant association between the type of specimen and cellularity; specifically, a specimen with cellularity score 3 has a 15 time higher probability of being ERM or PVR than ILM (odd ratio 15.0, 95% CI: 1.2–185.2). The relationship between the three cellularity score subgroups of ERM and patient's age was evaluated. The normality test highlighted normal distribution of patients' age for each cellularity score subgroup (score 1, *P*=0.99; score 2, *P*=0.90; and score 3, *P*=0.68). Significant differences in patients' age between the three subgroups of the cellularity score (*P*=0.124) was found: specifically, between subgroups of patients having their samples scored 1 and 2 (*P*=0.0127) and 1 and 3 (*P*=0.0304). No significant difference in patient's age was found between subgroups of patients having their membrane samples scored 2 and 3. In a summary, the analysis showed that the cellularity was related to the age; the younger the patient, the higher the cellularity score.  Figure 3 shows a boxplot of patient's age grouped by the cellularity score assigned to their membranes.

#### 3.1.1. Pigmentation

**Figure 4:**
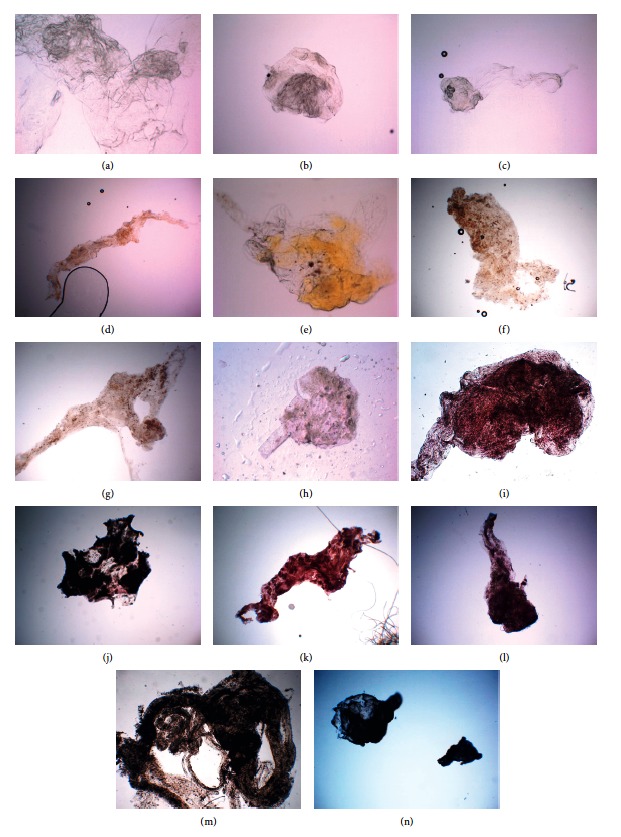
Pigmentation score of different specimens analysed in the study. Membranes from a macular pucker with pigmentation score 0: conventional epiretinal membrane (a); conventional epiretinal membrane removed with the internal limiting membrane (ERM + ILM) (b); internal limiting membrane (ILM) (c). (d, e) Atypical epiretinal membrane (A ERM) associated with lamellar macular hole (LMH) with pigmentation score 1. Specimens of membranes from diabetic retinopathy (DR) with score 1‐2: ERM from DR (f); ERM + ILM secondary to DR (g); ILM from ERM secondary to DR with pigmentation score 1 (h). Specimens with pigmentation score 3: ERM from RD with FTMH pigmentation score 3 (i); ERM from RD after PPV + PDMS with pigmentation score 3 (j); ERM associated with retinoschisis with pigmentation score 3 (k). (l–n) Proliferative vitreoretinopathy (PVR) specimens with pigmentation score 3.

**Figure 5:**
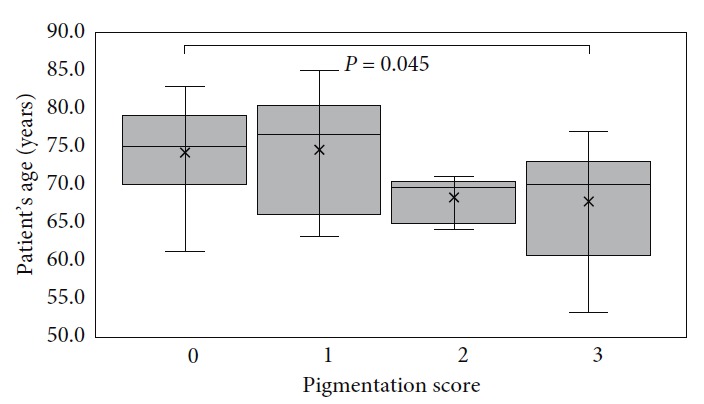
Pigmentation score boxplot vs patient's age. *P* value is indicated for subgroups showing statistical difference.

The pigmentation score was evaluated on 56 (79.7%) out of the 69 collected specimens (Figure 2). C ERM (8 specimens), C ERM + ILM (16 specimens), and ILM (11 specimens) associated with idiopathic MP were characterized by the absence (score 0) of pigmentation (Figures 4(a)–4(c)). The 2 specimens of A ERM associated with LMH were characterized by some degree (score 2) of pigmentation (Figures 4(d) and 4(e)). ERM (2 specimens), ERM + ILM (2 specimen), and ILM (5 specimens) associated with diabetic retinopathy were characterized by low-moderate (score 1 or 2) degree of pigmentation (Figures 4(d), 4(f)–4(h)). ERM secondary to RD repair under PDMS (1 case), ERM associated with RD and FTMH (1 case), and ERM associated with retinoschisis (1 case) were characterized by high degree of pigmentation (score 3) (Figures 4(i)–4(k)). PVR specimens were characterized by high degree of pigmentation (score 3) (Figures 4(l)–4(n)). We evaluated the association between pigmentation score and type of specimens, and a significant association between PVR specimen and pigmentation score 3 was found (Fisher's test *P* < 0.0001). The relationship between the three pigmentation score subgroups and patient's age was evaluated. Normality test highlighted normal age distribution for the four scores subgroups (score 0, *P*=0.12; score 1, *P*=0.71; score 2, *P*=0.13; and score 3, *P*=0.22). Significant difference on patients' age among the 4 subgroups of the pigmentation score (*P*=0.016) was found. The younger the patient, the greater the pigmentation score (Figure 5). Specifically, a significant difference between subgroup score 0 and score 3 was found (*P*=0.045).

#### 3.1.2. Scanning Electron Microscopy: Morphological Findings

**Figure 6:**
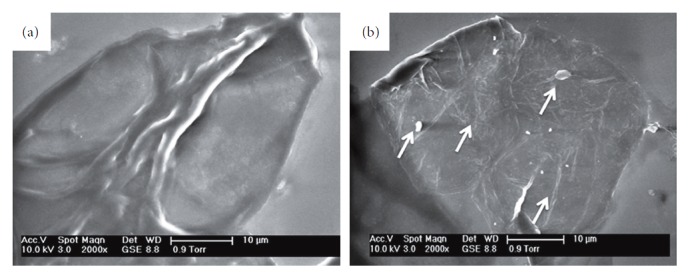
Scanning electron microscopy (SEM) of the vitreous (a) and retinal (b) surface of the internal limiting membrane (ILM). E-SEM mode, original magnification 2000 times.

**Figure 7:**
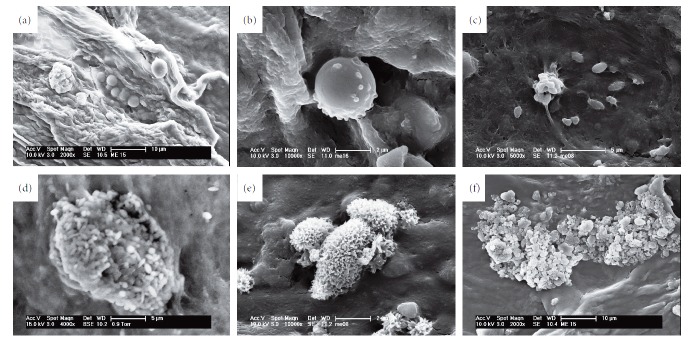
Morphological characteristics of adherent cells to the membranes surgically excised. (a, b) Rounded cells with a hole/indentation along the course of the collagen fibres, from the internal limiting membrane (ILM) specimen. (c) Rounded cells with short and long microvilli, from the epiretinal membrane (ERM) specimen. Elongated cells with regular (d) and irregular (e) villi from the proliferative vitreoretinopathy (PVR) specimen, corresponding to retinal pigmented epithelium (RPE) cells. (f) Globular arrangements of irregularly shaped granules and of microgranules with ovoid and needle shape. Scanning electron microscopy, HV-SEM mode, original magnifications ranging from 4000 to 10000 times.

ILM specimens were characterized on two different surfaces: vitreous side and retinal side. The retinal side appeared to be characterized by folds and cells corresponding to nerve fibres and retinal vessels. The vitreous side appeared smoother and more regular at the E-SEM investigation (Figures 6(a) and 6(b)). High-resolution magnification in the HV-SEM mode allowed to highlight the morphological characteristics of the cells adherent to the surgically excised membranes. On the ILM specimens, rounded cells with a hole/indentation along the course of the collagen fibres and a micromorphology compatible with the endfeet of the Müller cells were found at the HV-SEM investigation (Figures 7(a) and 7(b)). On the ERM specimens and typically on the C ERM associated with LMH, rounded cells with short and long microvilli (possibly myofibroblast-like cells) were found (Figure 7(c)). On the PVR specimens and A ERM associated with LMH, SEM showed elongated cells with irregular villi, with a morphology compatible with that of the RPE cells at different transdifferentiation stages (Figures 7(d) and 7(e)). Globular arrangements of microgranules with irregularly shaped granules (Figure 7(f)) were also identified, possibly related to the presence of pigmented aggregates.

#### 3.1.3. Immunohistochemical Study

**Figure 8:**
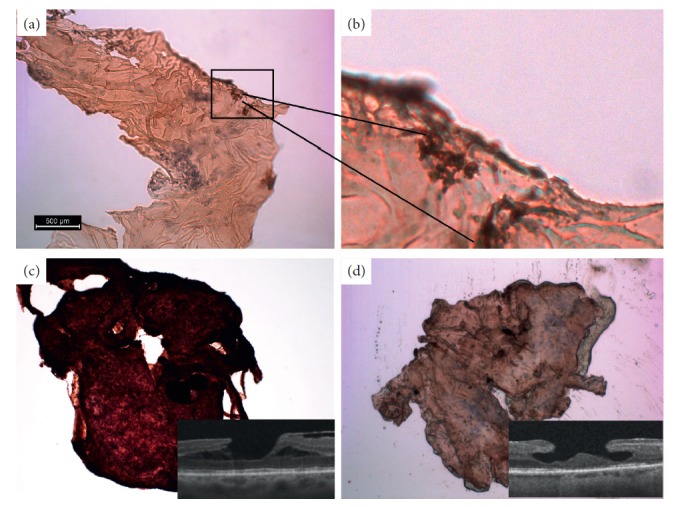
Immunohistochemical staining for CD68 or anti-*α*-SMA. (a, b) CD68 staining of epiretinal membrane (ERM) specimens. Macrophages appear as brownish agglomerate. (c, d) Anti-*α*-SMA staining of epiretinal membranes (ERM) associated with lamellar macular hole (LMH). Conventional ERM (c) appears brown due to the positive stain compared to the atypical epiretinal membrane (A ERM) (d). OCT images are given in the insets.

Positive staining to the macrophages marker (CD68) was found in few specimens of ERM (Figures 8(a) and 8(b)). Positive staining to anti-alpha-SMA was found in specimens of iERM and in a specimen of C ERM associated with LMH in which myofibroblast-like cells were identified. On the contrary, low-positive staining or no staining to anti-*α*-SMA was found in a specimen of A ERM associated with LMH (Figures 8(c) and 8(d)).

## 4. Discussion

### 4.1. Internal Limiting Membrane

**Figure 9:**
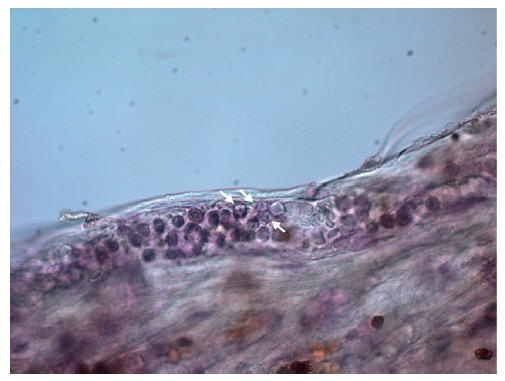
Polymorphonuclear leukocytes in the proliferative diabetic retinopathy (PDR) specimens, characterized by the nucleus usually lobed into three segments (white arrows). Original magnification: 640x.

The origin of the adhesion forces between the retina and the vitreous body is not yet fully understood, and little is known about the role of the ILM in maintaining this adhesion. Some studies report the presence of collagen fibres that from the vitreous cortex fit into the ILM's structure [20–24]. Vitreous cortex-ILM adhesion is age-related, and it thickens in the course of life, affecting the ability of Müller cells to maintain vitreomacular adhesion [15, 16, 25]. It has been recognized that SEM imaging allows to identify the ILM and ERM structures [26]. In the current paper, the authors found that ILM specimens were characterized by some degree of cellularity. ILMs were expected to be acellular, constituted by fibronectin, type IV collagen, and laminin, but the presence of cell components was detected by both OM and SEM on the surgically excised samples collected in this study. High-magnification SEM observation allowed to morphologically evaluate these cell components that appeared as vesicular structures adherent to the retinal surface of the ILM. These observations are confirmed by the experiments on the donor eyes published by Sebag [20], who associated these cell components to the endfeet of Müller cells by using transmission electron microscopy. However, there are important limitations in understanding how and why the proliferative vitreoretinal membranes develop. Currently, limited knowledge of the cells that are really involved in these processes is available. Some authors have used immunohistochemical staining using the glial fibrillary acidic protein (GFAP), a specific marker of Müller cells, detecting a strong positivity and concluding that Müller cells can proliferate and activate, thus becoming the main cells responsible for the formation of these membranes [18, 27–30]. Conversely, other studies have shown that GFAP positive cells also respond to markers of hyalocytes and macrophages, thus raising doubts about colocalization issues and the possibility of a reliable identification of these cells based on the sole immunohistochemistry. According to the latest available evidence, the most reliable hypothesis is that the hyalocytes are able to differentiate into macrophages and can phagocyte the endfeet of the Müller cells acquiring their specific markers [5, 15, 27, 28]. SEM images collected in this study allowed to highlight that the positivity of GFAP does not necessarily indicate the presence of Müller cells but could also be due to the presence of Müller cells endfeet alone, having no role in the development of the membrane. In addition to these considerations, the risk of iatrogenic damage of Müller cells due to the surgical removal of ILM should be considered as previously reported by other authors [25, 27]. ERM is a type of glial scar tissue produced in an attempt to protect the neuroretina from further damage by pathogenic factors present in the vitreous and injured RPE. ERM is composed of the extracellular matrix (collagen, laminin, tenascin, fibronectin, vitronectin, and thrombospondin) and other types of not fully identified cells. In this study, the cellularity of ERM specimens resulted to be inversely correlated with patient age. The higher the ERM cellularity, the lower the patient's age. This result could be influenced by the onset of the pathology, unknown to the authors, and it could express a transition from an initial cell proliferation phase to a phase of fibrotic evolution in the older age. The cells were not homogeneously distributed, but they appeared in clusters in different areas of the ERM specimens. In some cases, the authors identified the presence of macrophages into the cellular clusters. This finding could be an expression of an early stage of the evolution of ERM, an expression of active cell proliferation and cell repair, before the ERM evolution changes from early cellular composition to a late paucicellular and more fibrotic composition. Other studies confirmed that ERM was composed of glial cells and macrophages in the early stage, and then, over time, cellular component disappeared and ERM was eventually constituted by the extracellular matrix [31]. The pigmentation tissue score of ERM specimens in this study was variable. Tissue pigmentation was markedly higher in ERM specimens secondary to RD and in PVR. It was moderately present in A ERM with LMH and ERM associated with RDP. Pigmentation was completely absent in iERM. The pigmentation of ERM secondary to RD and PVR can be explained by the fact that RPE cells migrate through a retinal tear and reach the vitreoretinal interface [32–35]. The pigmentation of A ERM could be due to a migration of RPE cells through the intraretinal defect of the LMH. This hypothesis is supported by the study of Son et al. that observed positive pan-cytokeratin staining in the ERM associated with LMH [35]. Pan-cytokeratin is a monoclonal antibody of all human epithelia. RPE is the only layer of the retina that presents positivity to pan-cytokeratin staining. In a specimen of A ERM, a RPE-like cell was found, characterized by irregular microvilli, and no myofibroblasts were detected by anti-alpha-SMA. The authors suppose that RPE cells could migrate toward the vitreoretinal interface and transform in fibroblasts. This can explain the pigmentation and the absence of tractional properties of A ERM. There are similar theories that explain the presence of pigment into the ERM: other cells could undergo transformation into RPE cells, or RPE cells could migrate through occult breaks into the inner retina or through the inner retina stimulated by a defect of the ILM [28–38]. The reason for the presence of pigmentation in the ERM secondary to RDP remains still unclear. The pathological changes occurring during RDP are not fully understood yet. Haematoxylin staining allowed to identify the polymorphonuclear leukocytes in ERM associated with PDR because of the specific multinucleated morphology (Figure 9). Elevated systemic neutrophil count is associated with the presence and severity of DR as well as diabetes. This result indicates that systemic subclinical inflammation is related with DR, and neutrophil-mediated inflammation may play an important role in the pathogenesis of DR [39]. Regarding the staining with the alpha-SMA antibody in the C ERM and A ERM, the positivity was more frequently demonstrated in C ERM than in A ERM. Alpha-SMA is an intracellular actin presumed to be essential for extracellular matrix contraction [15]. The content of alpha-SMA in ERM was demonstrated to be correlated with clinical contractility [40, 41]. Cells of glial origin were shown to lose their typical GFAP expression by undergoing myofibroblast-like transdifferentiation, thereby losing the intermediate filament GFAP with coincident gains of SMA immunoreactivity [39].

## 5. Conclusions

OM and SEM proved to be informative tools for studying cell morphology of retinal membranes and represents a valid support for a deeper understanding of the available immunohistochemical study results. Indeed, the mutation in the expression of markers due to the transdifferentiation of the cells involved in these processes and the colocalisation of different markers do not allow the sole immunohistochemistry to give reliable information on the origin and identity of the cells involved in vitreoretinal proliferative processes. In this study, the authors highlighted a reduction of the cellularity of ERM related to the patients' age supporting the evolution of the ERM from a cell proliferative phase to a fibrous transformation. The detection of myofibroblasts demonstrates their main role in the development of ERM. The detection of pigment and RPE cells in A ERM specimens associated with LMH and in the secondary ERM specimens supports the hypothesis of a pathogenic role of RPE cells. Further studies integrating morphological investigation with immunohistochemistry on larger numbers of samples should be developed to unravel the type and role of different cells into the setting and development of vitreoretinal pathologies associated with proliferative membranes.

## Figures and Tables

**Table 1:** Membrane specimen subgroup and patient's demographic data.

Specimen subgroup	Total number of collected specimens	Patient age	Gender	Eye
	Number (%)	Years, mean ± SD (range)	Male/femalenumber	Right/left number
ILM	20 (28.9)	72.45 ± 5.78 (64–80)	9/11	11/9
ERM	22 (31.8)	73.22 ± 6.78 (58–88)	7/15	12/10
ERM + ILM	20 (28.9)	74.42 ± 7.01 (61–85)	10/10	12/8
PVR	7 (10.4)	63.57 ± 6 (53–70)	4/3	4/3

A ERM, atypical epiretinal membrane; C ERM, conventional epiretinal membrane; ILM, internal limiting membrane; PVR, proliferative vitreoretinopathy.

**Table 2:** Underlying eye pathology according to the membrane subgroup.

Pathology	ILM	ERM		ERM + ILM	PVR
		C ERM	A ERM		
	Number	Number	Number	Number	Number
Idiopathic MP	12	11	—	18	—
ERM secondary to CRVO	4	—	—	—	—
RD under PDMS	—	1	—	—	5
RD secondary to PVR	2	—	—	1	1
DR	1	6	—	—	—
RD secondary to FTMH	—	1	—	—	1
CNV	—	1	—	—	—
LMH	1	—	2	1 (A ERM + ILM)	—

A ERM, atypical epiretinal membrane; C ERM, conventional epiretinal membrane; ILM, internal limiting membrane; PVR, proliferative vitreoretinopathy; MP, macular pucker; CRVO, central retinal venous occlusion; RD, retinal detachment; PDMS, polydimethylsiloxane; DR, diabetic retinopathy; FTMH, full thickness macular hole; CNV, choroidal neovascularization; LMH, lamellar macular hole.

## Data Availability

The data used to support the findings of this study are included within the article.
